# Diversity and Characteristics of the Oral Microbiome Associated with Self-Reported Ancestral/Ethnic Groups

**DOI:** 10.3390/ijms252413303

**Published:** 2024-12-11

**Authors:** Qingguo Wang, Bing-Yan Wang, She’Neka Williams, Hua Xie

**Affiliations:** 1 School of Medicine, Meharry Medical College, Nashville, TN 37208, USA; 2 School of Dentistry, University of Texas Health Science Center at Houston, Houston, TX 77504, USA; bing-yan.wang@uth.tmc.edu; 3 School of Dentistry, Meharry Medical College, Nashville, TN 37208, USA; swilliams22@mmc.edu

**Keywords:** oral microbiome health disparities, periodontitis

## Abstract

Periodontitis disproportionately affects genetic ancestral/ethnic groups. To characterize the oral microbiome from different genetic ancestral/ethnic groups, we collected 161 dental plaque samples from self-identified African Americans (AAs), Caucasian Americans (CAs), and Hispanic Americans (HAs) with clinical gingival health or biofilm-induced gingivitis on an intact periodontium. DNA was extracted from these samples, and then DNA libraries were prepared and sequenced using an Illumina NovaSeq high-throughput sequencer. We found significant differences in the diversity and abundance of microbial taxa among dental plaque samples of the AA, CA, and HA groups. We also identified unique microbial species in a self-reported ancestral/ethnic group. Moreover, we revealed variations in functional potentials of the oral microbiome among the three ancestral/ethnic groups, with greater diversity and abundance of antibiotic-resistant genes in the oral microbiome and significantly more genes involved in the modification of glycoconjugates and oligo- and polysaccharides in AAs than in CAs and HAs. Our observations suggest that the variations in the oral microbiome associated with ancestral/ethnic backgrounds may directly relate to their virulence potential including their abilities to induce host immune responses and to resist antibiotic treatment. These finding can be a steppingstone for developing precision medicine and personalized periodontal prevention/treatment and for reducing oral health disparities.

## 1. Introduction

Periodontitis is a prevalent human disease affecting approximately 42% of adults aged 30 and older in the US. The National Health and Nutrition Examination Survey (2009–2014) highlighted significant ancestry and ethnicity disparities in periodontitis, with higher rates observed in African Americans (AAs, 56.6%) and Hispanic Americans (HAs, 59.7%) compared to Caucasian Americans (CAs, 37.0%) [1]. These disparities are influenced by multiple factors, including health care access and socioeconomic status (SES) [2,3]. A growing body of evidence shows that the human microbiome is composed of dynamic microbial communities that shift with states of health [4]. Microbiome dysbiosis has been identified in a long list of diseases using meta-omic techniques, including increased Staphylococcus aureus in the skin of atopic dermatitis [5] and decreased diversity and increased Fusobacteria in the gut microbiome in patients with colorectal cancer [6]. Interindividual differences in the microbiome are also influenced by genetics, environment, social determinants, and lifestyles [7,8], which leads to distinctive susceptibilities of populations with different ancestral/ethnic backgrounds to diseases. A recent study demonstrated a critical developmental window of gut microbiome variation at or shortly after 3 months of age, which is driven by social status and environments and may lead to health disparities in adults [9]. While oral microbial dysbiosis is a recognized factor in oral diseases [10,11], there remains a gap in understanding how variations in the oral microbiome contribute to the disparities. Recent studies reported a differential oral microbiota in the populations with oral malignant disorders and oral cancer when compared to that found in healthy counterparts [12,13,14]. Although signatures of the oral microbiome associated with oral cancer have not yet been established, some bacterial taxa, including *Fusobacterium*, *Prevotella*, and *Porphyromonas*, were more abundant in the oral cavities of patients with oral cancer. A population-level analysis of oral microbiome variation was also investigated in ancestral and ethnic groups. A study by Yang et al. found a significant ancestry difference in the oral microbiome between African ancestry and European ancestry [15]. The study of deep sequencing of 16S rRNA genes revealed a significantly increased abundance of four periodontitis-associated bacteria, including *Porphyromonas gingivalis*, *Prevotella intermedia*, *Treponema denticola*, and *Filifactor alocis*, in mouth rinse samples of African ancestry, compared to those in the samples from European ancestry, although the periodontal status of the cohort in this study is not known.

We previously investigated microbiologic risk factors associated with periodontal health disparities using qPCR. We determined and compared several key members of oral bacteria that play distinct roles in periodontal health in AAs, CAs, and HAs. We detected much higher levels of *P. gingivalis* in the plaque samples from the HA and AA periodontitis patients than in samples from the CA patients, which appears to be linked to a higher index of bleeding on probing observed in the HAs and AAs with periodontitis [16]. In addition, we examined the *P. gingivalis* level in the oral cavities of intact periodontium individuals with different ancestral/ethnic backgrounds. Although significant difference of *P. gingivalis* distributions in these groups was not found using qPCR in our previous study, the levels of *P. gingivalis* were higher in the HAs than that in the CAs [17]. To further identify population-associated differential microbial profiles, we selected a total of 161 dental plaque samples from AAs, CAs and HAs with intact periodontium for whole-metagenome shotgun sequencing that is a relatively more powerful approach for understanding diverse oral microbial communities and their functions. We identified a significant difference in the numbers of non-redundant bacterial genes and in the diversity and abundance of microbial species among the AA, CA, and HA groups. Additionally, several bacterial species were unique in a particular ancestral/ethnic group. Moreover, functional potentials of the oral microbiome, such as antibiotic resistance and LPS production, were higher in the AA group than in the CA and HA groups. These results suggest that the ancestry-/ethnicity-specific oral microbiome contributes to oral health disparities.

## 2. Results

### 2.1. Baseline Characteristics of Cohort

In previous studies, we determined the detection rates of *P. gingivalis*, using qPCR, among 340 individuals with intact periodontium with different ancestral/ethnic backgrounds [17]. The detection rates of *P. gingivalis* were 21.2% in AAs, 18.2% in CAs, and 30.6% in HAs. To further investigate variations in the oral microbiome associated with periodontal health disparities, we selected dental plaque samples from 44 AAs (GpgA), 42 CAs (GpgC), and 75 HAs (GpgH) (Table 1). GpgA, GpgC, and GpgH were further divided based on the absence (Gpg1A, Gpg1C, and Gpg1H) or presence (Group2A, Gpg2C, and Gpg2H) of *P. gingivalis* determined previously by qPCR [17]. Statistically significant differences were found in age and gender between the AA and HA groups and between the CA and HA groups, but not between the AA and CA groups (Table 1). Higher levels of BOP were found in AAs, compared to CAs and HAs (Negative Binomial Regression, *p* = 0.003 after adjusted for covariates including age and gender.

### 2.2. Diversity and Similarity of the Oral Microbiota Among AA, CA, and HA Groups

**Figure 1:**
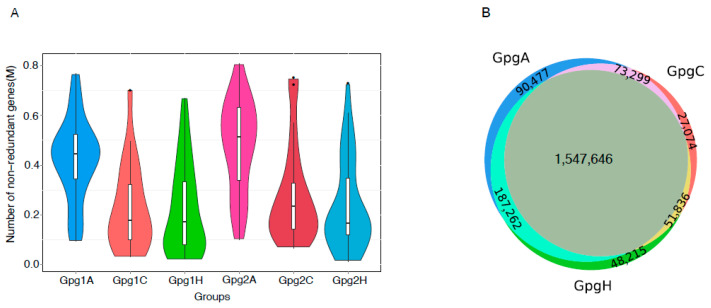
Comparison of the number of non-redundant genes in dental plaque samples from different ancestral/ethnic groups. (**A**) The violins represent the richness of non-redundant genes in the samples with low levels of *P. gingivalis* in AAs (Gpg1A), CAs (Gpg1C), and HAs (Gpg1H) or high levels of *P. gingivalis* in AAs (Gpg2A), CAs (Gpg2C), and HAs (Gpg2H). (**B**) Venn diagram of the total number of non-redundant genes identified in the dental plaque samples from AAs (GpgA), CAs (GpgC), and (GpgH).

A total of 2,025,809 non-redundant genes were predicted from 161 dental plaque samples using MetaGeneMark. As shown in Figure 1 and Table 2, the median number of non-redundant genes per sample in the AA groups were significantly higher than those found in their CA and HA counterparts (Negative Binomial Regression, *p* < 0.001) after adjusting for age and gender. There was no significant difference in the number of non-redundant genes between CAs and HAs, regardless of *P. gingivalis* detection with qPCR. In addition, we identified genes unique to each genetic ancestral/ethnic group (referred to here as ‘unique genes’). We found the highest number of unique genes (90,477) in the AA group followed by those (48,215 and 27,074) in HAs and CAs, respectively (Figure 1B). These finding suggest a more complex microbial composition in the AA population compared to HAs and CAs, which was also confirmed by taxonomic diversity among these groups.

**Figure 2:**
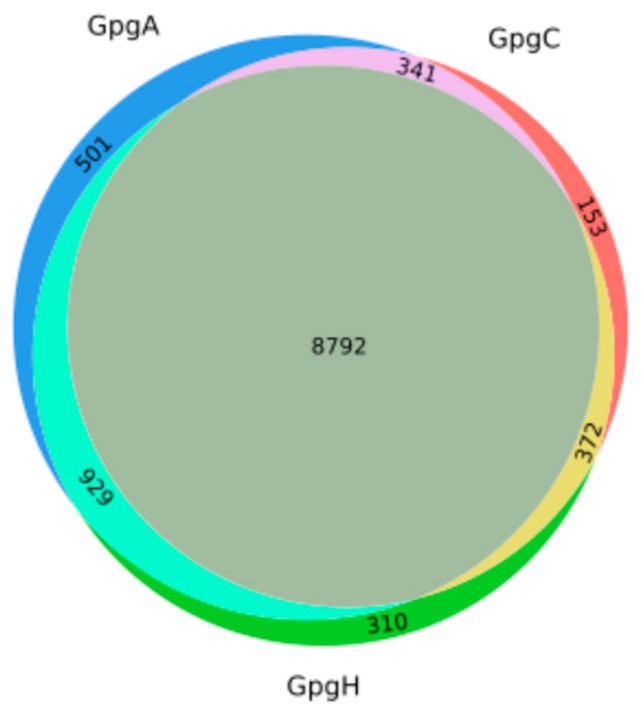
Venn diagram of the microbial taxa at the species level identified in the genetic ancestral/ethnic groups for AAs (GpgA), CAs (GpgC), and HAs (GpgH).

A total of 11,398 microbial species was identified in the 161 dental plaque samples using sequence or phylogenetic similarity to microNR. An average of more than 3000 species were predicted in each dental plaque sample from AAs, which was significantly higher than those found in the samples from CAs and HAs (Table 3). In addition, distinctive microbial species were identified in AAs, CAs, and HAs, which is consistent with the findings of unique genes among the groups. There were 501 microbial species found only in the AA group compared to 153 and 310 detected in the CA and HA counterparts, respectively (Figure 2). Table 4 presents the genetic ancestry-/ethnicity-specific species that were detected in five or more samples of each particular genetic ancestral/ethnic group. Interestingly, one bacterial species, *Pedobacter petrophilus*, was only detected in the samples from AAs with higher level of *P. gingivalis* (Gpg2A). These results imply that specific microbial profiles contribute to periodontal health disparities.

Notably, *P. gingivalis* was identified in all 161 samples with whole-metagenome shotgun sequencing even though *P. gingivalis* was detected in only 50% of the samples in this cohort using qPCR. However, samples (Gpg 2) in which *P. gingivalis* can be detected using PCR had a ten-fold higher abundance of *P. gingivalis* than those (Gpg 1) with *P. gingivalis* not detected using PCR (Table 5). Therefore, we re-designated Gpg 1 as the group with a low level of *P. gingivalis* and Gpg 2 as the high-level *P. gingivalis* group. In addition, *Tannerella forsythia*, *Fusobacterium nucleatum*, *Streptococcus cristatus*, and *Streptococcus gordonii* were also detected in all 161 samples. *Treponema denticola* and *Filifactor alocis* were identified in 160 and 158 samples out of 161 samples, respectively. We further determined the abundance of several well-studied bacterial species. We observed a significantly higher abundance of *P. gingivalis* and *T. denticola* in the dental samples retrieved from the AA and HA groups compared to the CA group (Table 6), whereas *F. alocis* and *T. forsythia* were detected significantly more in HAs than in CAs. We did not observe any significant difference in the abundance of *F. nucleatum* and *S. cristatus* among the three groups. We also found a significantly higher number of *S. gordonii* in AAs than in HAs, but not between AAs and CAs.

**Figure 3:**
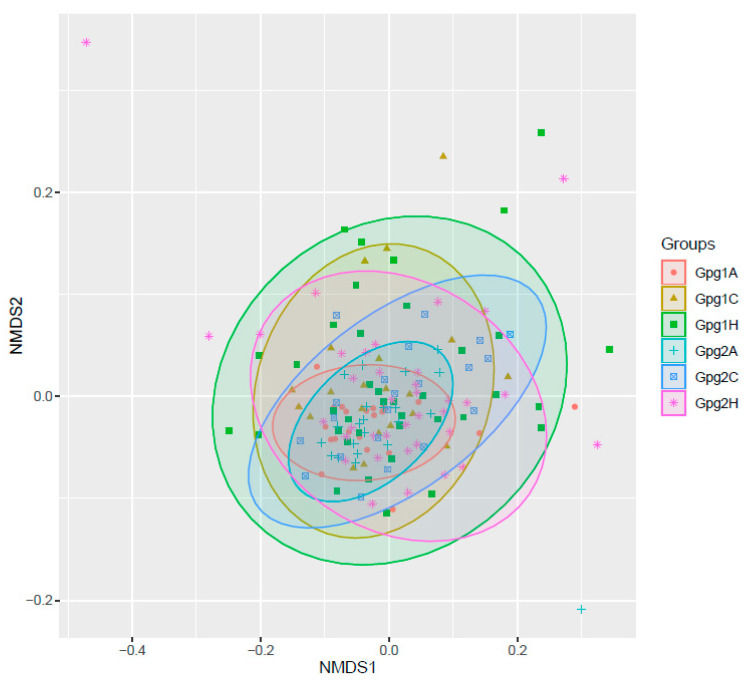
Visualization of NMDS analysis. Dots in a two-dimensional space represent dental plaque samples, and the distance between each pair of dots represents the dissimilarity between the corresponding two samples. The samples in the same groups were assigned the same colors.

To identify potential patterns of oral microbial profiles in different ancestral/ethnic groups, we projected the dental plaque samples from high-dimensional space into a two-dimensional space using NMDS, a non-supervised machine learning technique, after homogenizing the taxa abundance matrix. NMDS retains the original sample rank-order similarity by measuring it using Euclidean distance between the points that represent the samples. The visualization of the NMDS result is provided in Figure 3, which shows that the centers of the AA groups, Gpg1A and Gpg2A, are both located in the leftmost section of the figure in comparison with other group centers, whereas the centers of both Gpg1H and Gpg2H, i.e., the HA groups, are positioned further to the right in the figure, more away from the AA groups than the CA groups. Both Gpg1H and Gpg2H, i.e., the HA groups, are projected toward the bottom-right corner of the figure, further away from the AA groups than the CA groups. Interestingly, the Gpg1 groups (including Gpg1A, Gpg1C, and Gpg1H) occupy the upper section of the figure more than the Gpg2 groups (i.e., Gpg2A, Gpg2C, and Gpg2H), showing a distinction between the two groups which was determined based on the abundance of *P. gingivalis*. Moreover, the dots of Gpg1A and GpgA2 are much closer to one another than those of CAs and HAs (Figure 3), indicating that the microbial profiles are more similar among the samples in the AA groups than those of the other two groups.

### 2.3. Functional Diversity of the Oral Microbiome Associated with Genetic Ancestral/Ethnic Backgrounds

**Figure 4:**
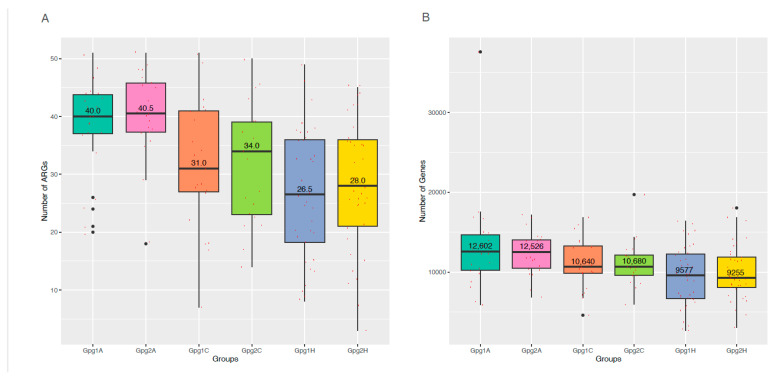
Diversity and abundance of antibiotic-resistant genes (ARGs). (**A**) Total number of ARG classes, and (**B**) the abundance of ARGs in each sample. Each sample is presented as a red point, and the number in each boxplot represents median number of ARG classes and abundance in a group.

**Figure 5:**
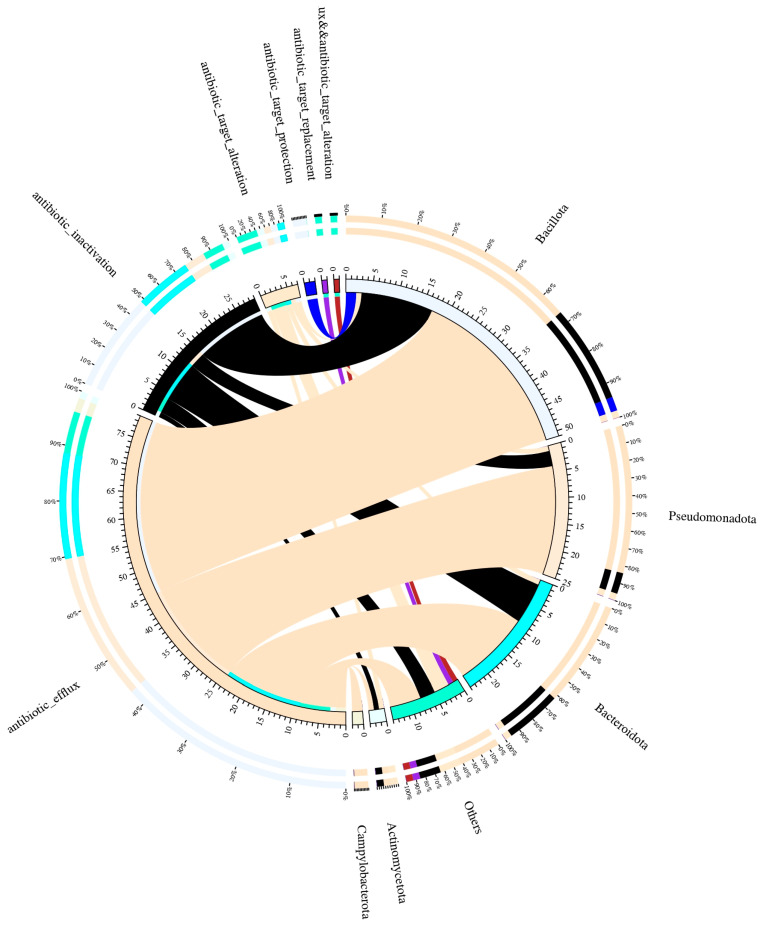
Antibiotic-resistant gene mechanism and the loop graph of species distribution. Circle chart is divided into two parts: the right side shows the sample information, and the left side shows the ARG tolerance of antibiotic information. Different colors in the inner circle represent different samples and ARGs and the scale for the relative abundance (unit ppm). The left side is the sum of the relative abundance of the resistance genes in the sample, and the right side is the sum of the relative abundance of the resistance genes in each ARG. The left side of the outer circle shows the relative percentage of the antibiotic to which the resistance gene belongs, and the right side of the outer ring shows the relative percentage of the sample in which the antibiotic-resistant gene is located.

Functional annotation was conducted by assembling metagenomic sequencing and mapping against functional protein databases. Mapping against the Comprehensive Antibiotic Resistance Database (CARD) allowed us to identify a total of 68 antibiotic-resistant genes (ARGs) in the 161 dental plaque samples (Figure 4A). The median numbers of ARGs per sample were 40 in the plaque samples from the AA group, 34 ARGs from the CAs, and 28 ARGs from the HAs. Additionally, more abundant ARGs (approximate medium copies of 12,600) were also detected in the samples from the AAs, compared to 10,600 and 9500 in the samples from the CAs and the HAs, respectively (Figure 4B). These data demonstrate that more complex and abundant ARGs were present in the oral microbiome of AAs compared to those in CAs and HAs, suggesting that the oral microbiomes of AAs may have a higher ability to resist antibiotics. Moreover, Figure 5 presents the functions of the ARGs. The majority of ARGs act on antibiotic efflux and inactivation, and others are involved in antibiotic target alteration, protection, and replacement. Most of the ARGs were found in *Bacillota*, a phylum of mostly Gram-positive bacteria, *Pseudomonadota,* including Gram-positive, Gram-negative, and variable bacterial species, and *Bacteroidota*, a phylum of Gram-negative bacteria (Figure 5), which presumably serve as reservoirs for ARGs in the oral microbiome.

**Figure 6:**
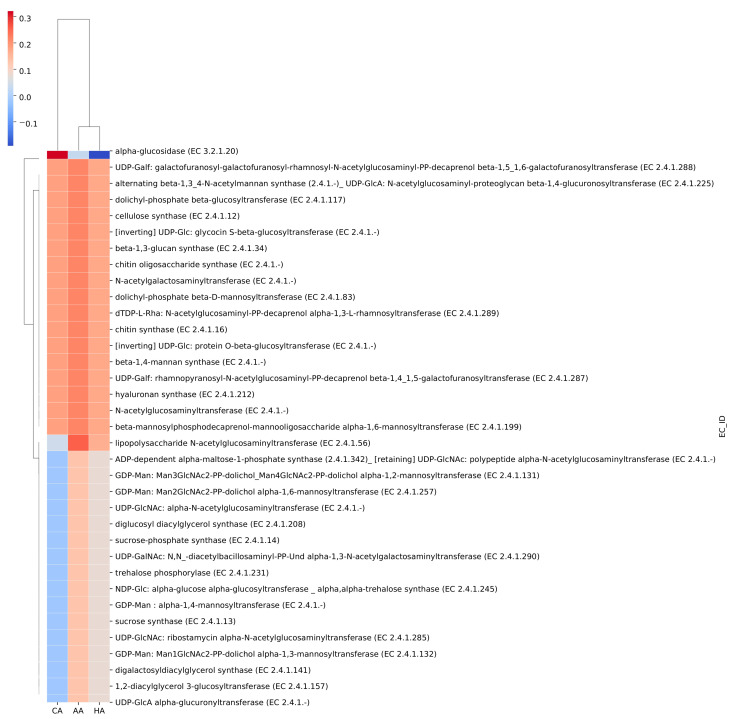
Heatmap of carbohydrate-active enzyme genes identified in AA, CA, and HA groups. Columns represent sample groups AA, CA, and HA, and rows represent genes. The red color represents high gene abundance to contrast with low abundance in blue color. The rows and columns are ordered based on the correlations of z scores, which were calculated based on gene abundance.

To determine the physiology of genetic ancestry-/ethnicity-associated oral microbiome, we also analyzed the assembled metagenomic protein sequences against the Carbohydrate-Active enZYmes Database (CAZy) [18]. A cluster heatmap shows the quantitation of the top 35 carbohydrate-active enzymes involved in the breakdown, biosynthesis, and modification of glycoconjugates and oligo- and polysaccharides (LPSs). The genes encoding these enzymes were much more abundant in the oral microbiome of the AAs, compared to those in the CAs and HAs (Figure 6). This implies that Gram-negative bacteria in the oral microbial communities may be relatively higher in the AA population than in CAs and HAs, which can induce immune-inflammatory responses and initiate the onset of periodontitis.

## 3. Discussion

In this paper, we investigated variations in the oral microbial communities across AA, CA, and HA groups using whole-metagenomic sequencing. Recent studies of the oral microbiome, which mainly used less powerful qPCR and 16S rRNA gene sequencing, found significant differences in the microbial compositions between/among genetic ancestral/ethnic populations, including a higher abundance of *P. gingivalis, T. forsythia, T. denticola*, and *F. alocis* in AAs [15,19,20,21]. Our results on the cohort with intact periodontium are consistent with previous reports that some well-known periodontal pathogens, including *P. gingivalis* and *T. forsythia,* were more abundant in AAs and HAs than in CAs. However, compared to previous qPCR-based studies that only detected *P. gingivalis* in approximately 25% of the samples from individuals without periodontitis [17,22], we found *P. gingivalis* in all the tested dental plaques in this study with whole-metagenomic sequencing, even though we previously did not identify *P. gingivalis* in half of the samples using qPCR. Moreover, *T. forsythia* was also found in all samples, while the detection rates of *T. denticola* and *F. alocis* were 99% and 98%, respectively. These results are not in agreement with some other studies that reported much lower detection rates of these bacteria using qPCR. For example, *T. forsythia* was detected in 10.5% of periodontally healthy participants and 68.0% of periodontitis patients. Moreover, our findings indicate that there is no difference in the detection rates of most well-known periodontal pathogens among ancestral/ethnic groups. These results indicate whole-metagenomic sequencing is more sensitive than qPCR and 16S rRNA sequencing for microbial detection at the species level of all taxa including viruses. Therefore, the detection rates of periodontal pathogens may not be closely associated with periodontal health disparities; rather, it is the levels of these pathogens in the oral microbiome that contribute to the initiation and development of periodontitis.

Of note, our detection rates for low-abundance *P. gingivalis* might be inflated by false-positive results, the filtering of which remains a challenge in the field. Although advancements in whole-metagenomic sequencing have improved both the sensitivity and the accuracy of computational pipelines, low-abundance taxonomies remain difficult to accurately characterize, because filtering out false positives is often complicated by contamination, sequencing artifacts, limited coverage for the taxa of interest, sequencing errors, and the ambiguities inherent in short-read mapping. Given the potential for false-positive and false-negative findings in metagenomic sequencing, a valuable future direction could be to incorporate multiple analytical pipelines and tools and use their consensuses to improve detection.

In addition, AAs, CAs, and HAs differ in microbial diversity. We identified higher numbers of microbial taxa in the oral microbiome of AAs and HAs than in CAs and greater diversity of microbial compositions in AAs and HAs than in CAs. Ancestry-/ethnicity-associated microbial species have not been reported to be associated with any human oral disease so far. Interestingly, we identified that some bacterial species were only found in the oral microbiota of AAs, such as *P. petrophilus*, which was clearly associated with higher levels of *P. gingivalis*. This bacterial species is currently not easily cultured in the laboratory, and hence it is difficult to analyze its relationship with other well-known periodontitis pathogens and its role in the initiating periodontitis. Nevertheless, the unique presence of this bacterial species in the oral cavities of the AA population may lead to a new research pathway linking to periodontal health disparities.

One advantage of whole-metagenomic sequencing is its ability to reveal information on functional potentials of the oral microbiome, enabling us to compare antibiotic resistance and carbohydrate enzyme activities of the oral microbiome among AAs, CAs, and CAs. Using metagenomic sequencing, we demonstrated more diverse and abundant ARGs in the oral microbiome of the AA group, which may be an important determinant of virulence that influences the ability of microorganisms to adapt and survive in oral cavities. It remains to be determined how complex and abundant ARGs evolve in the oral microbiome of the AA population. One possible mechanism is that inappropriate antibiotic use was more common among AA patients compared to CA patients [23]. Another unique characteristic of the oral microbiome of AAs is its higher level of enzymes involved in the modification of glycoconjugates and oligo- and polysaccharides, which may be linked to higher level of LPSs. This observation is consistent with the levels of several Gram-negative periodontal pathogens, including *P. gingivalis*, *T. forsythia*, *T. denticola*, and *F. alocis*.

In addition to the identification of functional potentials, other strengths of our study include a comprehensive periodontal examination for each participant and taxonomic annotation coverage at every taxonomic level including the species level. A previous study, using 16s rRNA sequencing, reported that African Americans had higher microbial diversity in oral washes than Caucasians [15]. By contrast, another study indicated that African American adults had the lowest bacterial diversity in subgingival plaques, while Chinese and Caucasian adults had the highest diversity [24]. Both studies lack detailed oral health parameters and used 16s rRNA sequencing. It should be pointed out that one limitation of this study is based on self-reported ancestry and ethnicity data. Two methods in health disparities were used to identify specific genetic ancestry and ethnicity, which include self-reported and genetic ancestry biomedical research. Each has its limitations [25]. However, previous studies suggested self-identified populations of AAs and CAs are generally reliable [26]. While the information derived from self-identified ancestry and ethnicity cannot explain precisely what genetic variables contribute to the differences among the populations, in the present study, we observed a significant difference in the oral microbiome among self-identified AA and CA populations, and relatively fewer differences were found between self-identified CAs and HAs, which may be due to the HA population having a more complex genetic makeup. Multiple factors, including genetic variable, socioeconomic status, education level, and diet, likely have an influence on the composition and function of the oral microbiome. For example, HAs have distinctive cultural characteristics, including diet, and it remains unclear which specific Hispanic food has effects on the oral microbiome. It was reported that some food additives, such as octanoic acid, decanoic acid, acesulfame K, aspartame, saccharin, and sucralose, were able to inhibit the growth of *P. gingivalis* [27]. Therefore, more accurate information on ancestry proportions and on traditional diets of the participants will be included in future health disparity studies. In summary, this study shows significant differences in oral microbial diversity and abundance among self-reported AA, CA, and HA groups. We also identified ancestry-/ethnicity-specific bacterial species, such as *P. petrophilus,* that was only found in AAs with relatively higher *P. gingivalis* levels. Moreover, higher functional potential, including antibiotic resistance and LPS production, were observed in the oral microbiome of AAs. These differences may shape the virulence potential of the oral microbiome and prepare the microbiome as a whole to adapt to their environmental niches. Understanding and comparing the ancestry-/ethnicity-associated oral microbiome provide a steppingstone for improving oral health disparities.

## 4. Materials and Methods

### 4.1. Study Cohorts

The research protocol was approved by the Committee for the Protection of Human Subjects of the University of Texas Health Science Center at Houston (IRB number: HSC-DB-17-0636). Candidates were screened during routine dental visits at the clinic of the School of Dentistry, University of Texas Health Science Center at Houston between 2017 and 2022. Individuals aged 21–75 years with self-reported ancestry of AAs, CAs, or HAs were enrolled after the initial periodontal examination that included determination of plaque index (PI), bleeding on probing (BOP) level, probing depth, and clinical attachment level on all teeth [28]. Radiographs were taken during this screening phase to assess bone loss. The clinical oral examinations were performed by faculty members of the School of Dentistry, University of Texas Health Science Center at Houston. The examiners are calibrated annually in the diagnosis of periodontitis. Based on the 2017 World Workshop classification [29,30], all study participants diagnosed with clinical gingival health or biofilm-induced gingivitis on an intact periodontium met the following criteria: >24 teeth; no alveolar bone loss or clinical attachment loss; pocket depth ≤ 3 mm (excluding pseudo pocket); no antibiotic therapy in the previous six months; and not pregnant.

### 4.2. Dental Plaque Sample Collection

Dental plaque samples, including supra- and subgingival dental plaques, were collected by board-certified periodontists using sterile paper points prior to any dental treatment and labeled numerically according to the sampling sequences. The paper points were placed in the sulci of all the first molar in different quadrants for 1 min and then immersed immediately in an Eppendorf tube with 0.5 mL of Tris-EDTA (TE) buffer (pH 7.5) [31]. Bacterial pellets were harvested by centrifugation and then resuspended in 100 µL TE buffer. Samples were stored at −80 °C until use.

### 4.3. Sequencing and Quality Control

Samples were sent to Novogene Co. (Sacramento, CA, USA) with dry ice for metagenomic sequencing. Briefly, DNA extracted from human dental plaques was randomly sheared into short fragments, and the resulting fragments were end-repaired, A-tailed, and ligated using Illumina adapters. The fragments with adapters were subsequently amplified using PCR, then size selected and purified. Quality control of the library was conducted using Qubit (≥20 ng, ≥10 ng/μL), and quantification and size distribution detection were performed using real-time PCR and a bioanalyzer, respectively. The quantified libraries were pooled and sequenced using an Illumina NovaSeq high-throughput sequencer by Novogene Corporation, Inc., with a paired-end sequencing length of 150 bp and an output of ~6 GB of raw data per sample.

### 4.4. Data Preprocessing

The average size of raw data generated per sample was 6.394 GB. To ensure accuracy and reliability of the subsequent data analysis, all low-quality bases (Q-value ≤ 38) that exceeded certain threshold (40 bp), as well as reads containing N nucleotides over 10 bp and those overlapping with adapters > 15 bp were trimmed. After quality control, the size of clean data was 6.393 GB per sample, with 96.36% and 91.26% of bases having quality scores greater than 20 and 30, respectively. To minimize host DNA contamination, raw reads that were mapped to the human reference genome hg38 were discarded using the Bowtie2 software (version 2.0) [32].

### 4.5. Gene Prediction and Abundance Analysis

The sequencing reads of samples were assembled using tool MEGAHIT into scaftigs (i.e., continuous sequences within scaffolds), which were filtered to remove those less than 500 bp in length. The effective scaftigs were then used for gene prediction and other analysis.

The MetaGeneMark software (version 2.10) was used to predict open reading frames (ORFs) from scaftigs (≥500 bp) [33]. ORFs less than 100 nt were discarded. To generate gene catalogs, the remaining ORFs were dereplicated using CD-HIT [34,35] in default settings (i.e., identity = 95% and coverage = 90%). To calculate the gene quantity, the clean data were mapped to the gene catalog using Bowtie2 (parameters: –end-to-end, –sensitive, –I 200, and –X 400). Gene abundance (*Gk*) was calculated using the following formula:

$$G_{k}=\frac{r_{k}}{L_{k}}\frac{1}{\sum_{i=1}^{n}\frac{r_{i}}{L_{i}}}$$

where *r* represents the number of mapped reads and *L* represents gene length. Downstream analyses were performed based on the abundance of the gene catalog.

Bacterial abundance is estimated through gene abundance and is normalized using fragments per kilobase of transcript per million mapped reads (*FPKM*). The *FPKM* normalizes read count based on species genome size *L* and the total number of mapped reads *N*. The formula for calculating *FPKM* for a species is as follows: *F* denotes the number of fragments mapped to the species; *L* is the length (in kilobases) of the species’ reference genome, and *N* is the total number of mapped reads across all species in that sample.

$$FPKM=\frac{F\times 10^{9}}{L\times N}$$

In addition to absolute abundance, e.g., *FPKM*, relative abundance of species in each metagenome is also used when comparing them across samples and groups. With relative abundance values often varying greatly between species and samples, we also computed their z scores to standardize these values, to make them visually interpretable in plots, such as heatmap in Figure 6.

### 4.6. Taxonomy Annotation

DIAMOND software (version 0.9.9.110) [36] was used to align the sequences of the identified genes to those of bacteria, fungi, archaea, and viruses extracted from NCBI’s NR database (version 2018-01-02). MEGAN software (version 6) was used to taxonomically annotate each metagenomic homolog [37]. The sum of abundance of genes annotated as a species in a sample was used as the abundance estimate of that species in that sample. Based on the abundance of each taxonomic level, various analyses were performed, including heatmap of abundance, principal coordinate analysis (PCoA), principal component analysis (PCA), and non-metric multi-dimensional scaling (NMDS) analysis, which is an indirect gradient analysis approach that produces ordination based on a distance matrix. R package ade4 (version 3.2.1) was used to perform PCA analysis, and R package vegan (version 2.15.3) was used for PCoA and NMDS analyses.

### 4.7. Functional Analysis

The identified gene sequences were aligned to those in functional databases utilizing DIAMOND software (version 0.9.9.110) [36], with parameter settings: blastp and -e 1e-5. The functional databases used in this study included Comprehensive Antibiotic Resistance Database (CARD) (version 3.2.6) [38], Carbohydrate-Active Enzymes Database (CAZy) (version 2023.03) [18], Kyoto Encyclopedia of Genes and Genomes (KEGG) (version 2024.03) [39], and Genealogy of Genes: Non-supervised Orthologous Groups (eggNOG) (version 5.0) [40]. Based on the sequence alignment results, the best Blast hits were selected for subsequent analysis and the relative abundance was calculated at different functional levels.

### 4.8. Statistical Analysis

Statistical analyses were conducted using statsmodels (version 0.10.1) and SciPy (version 1.4.1), open-source Python libraries for statistical modeling and scientific computing. Chi-square tests were performed to compare gender of different genetic ancestral/ethnic groups. Kruskal–Wallis tests were applied to compare ages of the sample groups. For the total number of non-redundant genes and taxa characterized in each sample, Negative Binomial Regression was performed to adjust for age and gender while comparing the three genetic ancestry groups. In addition, to compare the abundance of a particular species in the three genetic ancestry groups, linear regression was firstly performed to fit the data, adjusting for covariates (age and gender), and Kruskal–Wallis test was then applied to the residuals extracted from the regression model to calculate *p* values. A *p*-value of <0.05 was considered statistically significant.

## Figures and Tables

**Table 1:** Characteristics of the study cohort.

Characteristics	AAs	CAs	HAs	*p*-Value
Gender (Male/Female)	20/24	16/26	17/58	0.027 ^+^
Age (Year; Mean ± SD)	44.84 ± 2.84	48.63 ± 2.85	41.08 ± 12.58	0.029 ^#^
BOP (%, Mean ± SD)	34.92 ± 5.23	17.30 ± 3.11	22.53 ± 18.29	0.006 *
PI (%, Mean ± SD)	61.04 ± 5.87	50.12 ± 5.24	44.94 ± 25.10	0.093
Tooth number (Mean ± SD)	26.72 ± 0.47	27.04 ± 0.35	27.17 ± 2.22	0.963

BOP: bleeding on probing and PI: modified O’Leary plaque index. Tooth number is based on a total of 32 teeth. ^+^ The gender distribution of AAs is significantly different from that of HAs, and there is no significant difference between AAs and CAs or between CAs and HAs (Chi-square test). ^#^ The age distribution of AAs is significantly different from other two groups, which do not have a significant difference in ages (Kruskal–Wallis test). * The BOP of AAs is significantly different from those of CAs and Has, which do not differ significantly in BOP (Negative Binomial Regression, adjusted for covariates age and gender).

**Table 2:** Non-redundant genes identified per samples in different groups.

Comparison of Sample Groups	Median Number of Genes per Sample	*p*-Value *
Gpg1A vs. Gpg2A	445,282 vs. 513,094	0.596
Gpg1C vs. Gpg2C	178,020 vs. 235,183	0.371
Gpg1H vs. Gpg2H	171,843 vs. 167,841	0.884
Gpg1A vs. Gpg1C	445,282 vs. 178,020	4.34 × 10^−3^
Gpg1A vs. Gpg1H	445,282 vs. 171,843	1.86 × 10^−3^
Gpg1C vs. Gpg1H	178,020 vs. 171,843	0.862
Gpg2A vs. Gpg2C	513,094 vs. 235,183	1.38 × 10^−2^
Gpg2A vs. Gpg2H	513,094 vs. 167,841	6.92 × 10^−4^
Gpg2C vs. Gpg2H	235,183 vs. 167,841	0.295

* *p* values were calculated using the Negative Binomial Regression model after adjusting for covariance.

**Table 3:** The mean number of taxonomies identified per sample in different groups.

	Mean Number of Species	*p*-Value *
Gpg1A vs. Gpg2A	3184 vs. 3893	0.299
Gpg1C vs. Gpg2C	2028 vs. 2268	0.165
Gpg1H vs. Gpg2H	1836 vs. 2030	0.349
Gpg1A vs. Gpg1C	3184 vs. 2028	2.00 × 10^−3^
Gpg1A vs. Gpg1H	3184 vs. 1836	4.19 × 10^−4^
Gpg1C vs. Gpg1H	2028 vs. 1836	0.860
Gpg2A vs. Gpg2C	3893 vs. 2268	7.17 × 10^−3^
Gpg2A vs. Gpg2H	3893 vs. 2030	8.26 × 10^−5^
Gpg2C vs. Gpg2H	2268 vs. 2030	0.172

* *p* values were calculated using the Negative Binomial Regression model after adjusting for covariance.

**Table 4:** Unique bacterial species found in AAs and HAs.

	Numbers of Samples with the Unique Species				
	AAs			HAs	
Species	Gpg1A	Gpg2A *	Species	Gpg1H	Gpg2H
*Streptomyces* sp. *WAC04114*	2	3	*Micrococcales bacterium*	3	5
*Vagococcus hydrophili*	5	0	*Megasphaera* sp. *DISK 18*	2	4
*Veillonella* sp. *OK1*	4	2	*Ruminococcus* sp. *NSJ-71*	3	3
*Pedobacter petrophilus*	0	7	*Ideonella azotifigens*	1	4
*Hyphomicrobium* sp. *CS1GBMeth3*	1	5	*Pseudomonas caricapapayae*	4	2
*Halomonas zhangzhouensis*	3	2	*Siphovirus Jomon_CT89*	2	4

* Gpg1 included samples with the median abundance (FPKM) of *P. gingivalis* (<5741 FPKM), and Gpg2 had samples with the median abundance (FPKM) of *P. gingivalis* (>59,862 FPKM).

**Table 5:** Detection rates of well-known oral bacteria in different groups.

Sample Group	Gpg1A	Gpg1C	Gpg1H	Gpg2A	Gpg2C	Gpg2H	Gpg1	Gpg2
Sample count	22	21	38	22	21	37	81	80
*P. gingivalis*	22	21	38	22	21	37	81	80
*F. alocis*	22	20	37	22	21	36	79	79
*T. forsythia*	22	21	38	22	21	37	81	80
*T. denticola*	22	21	37	22	21	37	80	80
*F. nucleatum*	22	21	38	22	21	37	81	80
*S. cristatus*	22	21	38	22	21	37	81	80
*S. gordonii*	22	21	38	22	21	37	81	80

**Table 6:** The average abundance (FPKM) of species per sample in different groups.

Sample Group	Abundance			*p*-Value		
	GpgA	GpgC	GpgH	GpgA vs. GpgC	GpgA vs. GpgH	GpgC vs. GpgH
*Porphyromonas gingivalis*	159,821	82,068	112,880	0.002	0.015	0.021
*Filifactor alocis*	48,664	28,642	22,435	0.086	0.102	0.007
*Tannerella forsythia*	102,822	73,717	104,979	0.133	0.441	0.039
*Treponema denticola*	64,395	34,671	83,108	0.001	0.115	0.0003
*Fusobacterium nucleatum*	323,709	233,939	189,829	0.392	0.254	0.860
*Streptococcus cristatus*	26,989	28,667	29,825	0.749	0.908	0.712
*Streptococcus gordonii*	38,290	29,472	23,824	0.110	0.005	0.199

## Data Availability

Our metagenomic sequencing data were deposited at Sequence Read Archive (SRA) with accession number PRJNA1160290.
